# Ohio District Dental Societies

**Published:** 1889-04

**Authors:** 


### Ohio District Dental Societies.

At the last meeting of the Ohio State Dental Society a committee was appointed to take into consideration the matter of organizing district societies through the State under such an arrangement as would embrace the entire State; this committee was given power to act so far, as in its judgment, it was thought advisable. After pretty thoroughly canvassing the matter it was decided that such an organization was not only practicable, but eminently desirable, and would doubtless result in great good to the profession in the State. In the discharge of this duty the committee has proceeded to arrange the State into eight districts (giving to each ten or twelve counties) according to location and population, taking into account the distribution of members of the profession as well. Each district has been assigned a name by which its location will be indicated, and also a number, which for certain purposes will be more convenient than a name. The Central District, No. 1, occupies the central part of the State.

The other districts are indicated as follows, viz.: No. 2, the Northwestern; No. 3, the Northern; No. 4, the Northeastern; No. 5, the Eastern; No. 6, the Southern; No. 7, the Southwestern; No. 8, the Western.

A constitution and by-laws and rules of order have been drafted, which it is presumed will be adapted to the needs of these societies, or at least with but little modification. It would seem that the constitutions of these various societies should be as nearly uniform as possible. The profession in these various districts will be notified at an early day that the work of this committee has been about completed, and that the carrying out of the scheme now, in the main, rests with them.

It is to be hoped that at an early day the profession in each of these districts will take up the work and arrange for meetings for organization, and getting the work into active operation.

The committee consists of Drs. L. P. Bethel, of Toledo ; C. R. Butler, of Cleveland ; H. H. Harrison, of Cadiz ; A. F. Emminger, of Columbus, and J. Taft, Cincinnati, either of whom may be addressed for information on the subject. For maps, constitution, etc., address Dr. Bethel, Chairman of the Committee.

